# Deconvoluting binding sites in amyloid nanofibrils using time-resolved spectroscopy†

**DOI:** 10.1039/d2sc05418c

**Published:** 2023-01-19

**Authors:** Bo Jiang, Utana Umezaki, Andrea Augustine, Vindi M. Jayasinghe-Arachchige, Leonardo F. Serafim, Zhi Mei Sonia He, Kevin M. Wyss, Rajeev Prabhakar, Angel A. Martí

**Affiliations:** a Department of Chemistry, Rice University Houston TX USA amarti@rice.edu; b Department of Chemistry, University of Miami Coral Gables FL USA rpr@miami.edu; c Department of Bioengineering, Rice University Houston TX USA; d Department of Materials Science & Nanoengineering, Rice University Houston TX USA

## Abstract

Steady-state fluorescence spectroscopy has a central role not only for sensing applications, but also in biophysics and imaging. Light switching probes, such as ruthenium dipyridophenazine complexes, have been used to study complex systems such as DNA, RNA, and amyloid fibrils. Nonetheless, steady-state spectroscopy is limited in the kind of information it can provide. In this paper, we use time-resolved spectroscopy for studying binding interactions between amyloid-β fibrillar structures and photoluminescent ligands. Using time-resolved spectroscopy, we demonstrate that ruthenium complexes with a pyrazino phenanthroline derivative can bind to two distinct binding sites on the surface of fibrillar amyloid-β, in contrast with previous studies using steady-state photoluminescence spectroscopy, which only identified one binding site for similar compounds. The second elusive binding site is revealed when deconvoluting the signals from the time-resolved decay traces, allowing the determination of dissociation constants of 3 and 2.2 μM. Molecular dynamic simulations agree with two binding sites on the surface of amyloid-β fibrils. Time-resolved spectroscopy was also used to monitor the aggregation of amyloid-β in real-time. In addition, we show that common polypyridine complexes can bind to amyloid-β also at two different binding sites. Information on how molecules bind to amyloid proteins is important to understand their toxicity and to design potential drugs that bind and quench their deleterious effects. The additional information contained in time-resolved spectroscopy provides a powerful tool not only for studying excited state dynamics but also for sensing and revealing important information about the system including hidden binding sites.

## Abstract 2

Deconvolution of binding equilibrium data measured by time-resolved spectroscopy revealed two binding sites of a ruthenium complex when bound to amyloid-β fibrils: one elongated the lifetime of ruthenium complex and the other did not affect its lifetime.

## Graphical abstract

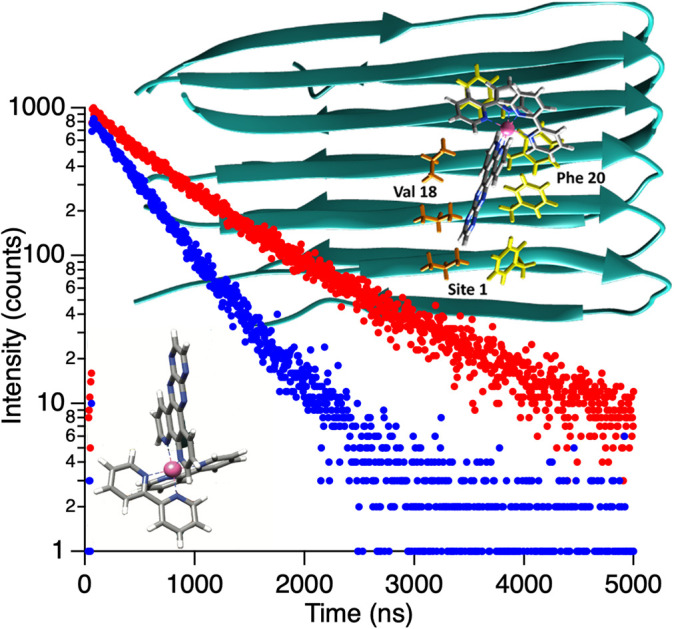

## Introduction

Alzheimer's disease (AD) is a degenerative disease that affects the human brain, particularly in elderly people, with symptoms that include memory loss, confusion, and personality changes.^1^ The amyloid cascade hypothesis proposes the accumulation of amyloid-β (Aβ) aggregates as a mechanism in the progression of Alzheimer's disease.^2,3^ While the relation between amyloid aggregates (soluble and insoluble) and the onset of AD has been known for more than 100 years, it was only last year when the first amyloid-targeting drug (an antibody called Aducanumab^4^) was approved by the FDA, with many other medications of the same kind on the horizon.^5^ This has prompted a resurgence of interest in small molecule-based treatments targeting amyloids. Understanding how small molecules bind and interact with amyloid aggregates is, therefore, essential to developing appropriate treatments for AD. The study of binding sites in amyloid aggregates has greatly benefited from the use of photoluminescence titration experiments. This method provides the dissociation constants of ligands by simply measuring the steady-state photoluminescence intensities over a series of samples. However, standard steady-state titration methodologies have the drawback that they assume the target molecule has a single unique binding site. Therefore, the presence of multiple binding sites needs to be confirmed through other analytical methods.^6^

A different method to study the photoluminescence of a molecular probe is time-resolved spectroscopy. Lincoln *et al.* reported that they observed two different lifetimes while studying the emission of ruthenium(ii) complex bound to DNA, which were yielded by two different geometries of binding to DNA.^7–9^ More relevant to amyloid proteins, fluorescence lifetime imaging microscopy (FLIM) has been used to image and study amyloid fibrils based on changes in lifetime.^10^ These studies have used lifetime in qualitative ways, not fully capitalizing on the reach of time-resolved spectroscopy. We have expanded the application of time-resolved spectroscopy to determine quantitative binding information on fibrillar amyloids. This also compensates for the shortfalls of steady-state photoluminescence titrations, particularly when photoluminescent metal complexes are used as probes.

We have previously reported the use of photoluminescent metal complexes of ruthenium^11–15^ and rhenium^16,17^ to study amyloid aggregates.^18,19^ Particularly, we investigated the binding of light-switching ruthenium and rhenium dipyridophenazine complexes to Aβ fibrils using steady-state photoluminescence titrations, molecular dynamics (MD) simulations,^11^ and photochemical footprinting.^16^ These studies have provided important information on the binding equilibrium of small molecules to Aβ, but the values were obtained under the assumption that Aβ has only one binding site. In another work, we used a ruthenium polypyridyl complex ([Ru(bpy)_2_(dpqp)]^2+^, bpy = 2,2′-bipyridine, dpqp = pyrazino[2′,3′:5,6]pyrazino[2,3-f][1,10]phenanthroline) to monitor the formation of Aβ oligomers using photoluminescence anisotropy.^15^ This metal complex was selected because it lacks a strong light-switching response in the presence of Aβ aggregates,^20^ which enable us to observe the anisotropy change of the complex. A molecule without the light-switching response is impractical for determining the binding information using steady-state photoluminescence titration; however, it is ideal for monitoring all the species present in a system (*e.g.* bound to Aβ fibrils and free in solution) using time-resolved spectroscopy.

Here, we study binding sites on Aβ using [Ru(bpy)_2_(dpqp)]^2+^ as a probe and time-resolved photoluminescence spectroscopy to evaluate the concentration of probes bound to Aβ. When performing titrations to elucidate the binding equilibrium, we found that two different binding sites are present, one that promotes a change in the lifetime of the ruthenium complex and the other that does not affect the lifetime. MD simulations were carried out to shed light on the nature of the interaction between [Ru(bpy)_2_(dpqp)]^2+^ and Aβ fibrils. By selectively deconvoluting the emission from [Ru(bpy)_2_(dpqp)]^2+^ bound to Aβ, we were able to monitor the aggregation of Aβ in real-time. The formation of Aβ fibrils was confirmed by atomic force microscopy (AFM). In addition, we demonstrated the binding of common ruthenium and iridium polypyridine complexes ([Ru(bpy)_3_]^2+^ and [Ir(ppy)_2_(bpy)]^+^, ppy = 2-phenylpyridine) to two different binding sites on Aβ fibrils. Interestingly, both [Ru(bpy)_3_]^2+^ and [Ir(ppy)_2_(bpy)]^+^ showed binding to Aβ, even when they do not have traditional Aβ recognition (or binding) elements. Due to the relevance of Aβ aggregation on the development of AD, elucidating new ways to study Aβ aggregates, their binding sites, and the process of aggregation is of great importance.

## Experimental section

### Purification of Aβ_1–40_

Synthetic Aβ_1–40_ was purchased from 21st Century Biochemicals. For HPLC purification, desalted Aβ_1–40_ was dissolved in 0.1% TFA in water by vortexing for 1 min. HPLC purification was performed using a reverse-phase C-18 peptide column with a gradient elution of water with 0.1% TFA–acetonitrile changing from 70 : 30 to 10 : 90 over 25 min with a flow rate of 1.5 mL min^−1^ at 60 °C. Purified Aβ was lyophilized and stored at −20 °C.

### Preparation of Aβ solutions

A basic stock solution of monomeric Aβ was prepared by dissolving one tube of purified Aβ in 200 μL of 26.5 mM NaOH solution. The solution was diluted to 400 μL, and the concentration was measured using a UV-Visible spectrometer (Shimadzu 2450) at 292 nm (*ε* = 2132 M^−1^ cm^−1^). The stock Aβ solution was diluted to a final concentration of 100 μM by adding phosphate buffer (PB). The final concentration of PB was 25 mM in the Aβ solution, and the pH was set at 7.4. For fibrils formation, the solution was incubated at 37 °C, with orbital stirring at 600 rpm. To quantify the amount of non-fibrillar species left after incubation, the incubated mixture was passed through a 10 kDa filter (Amicon Ultra-0.5 Centrifugal Filter Devices) at 14 000 g for 15 minutes. The absorbance of the filtered solution was compared to the absorbance of a control sample of a non-incubated buffered solution of Aβ, both measured at 280 nm. The non-fibril components of the final incubated solution was determined to be *ca.* 6% of the starting concentration of Aβ monomers. Therefore, all calculations of Aβ fibril concentrations were adjusted by a correction factor of 94%.

### Synthesis of [Ru(bpy)_2_(dpqp)]^2+^

The synthesis of this compound is reported elsewhere.^20^ Briefly, *cis*-Ru(bpy)_2_Cl_2_ and the dpqp ligand were refluxed for 4 h in a mixture of 1 : 1 ethanol/water. After the mixture reached room temperature, the volume of the mixture was halved, and then a saturated aqueous KPF_6_ solution was added to precipitate the PF_6_ complex. The purification of this compound was performed by successive precipitations from acetone/KPF_6_ (aq), where the aqueous soluble phases were extracted with dichloromethane (3 × 10 mL). The solution was evaporated to dryness, and the solid was washed with diethyl ether, yielding a red solid attributed to [Ru(bpy)_2_(dpqp)](PF_6_)_2_ (73% yield). To make the complex soluble in water, tetrabutylammonium chloride was mixed with the PF_6_ complex in acetone, converting the product to the chloride salt.

### Binding assay (saturation titration experiments)

A constant 4 μM concentration of the metal complex was mixed with different concentrations of Aβ fibrils (2, 5, 15, 20, 30, 40, 60, and 80 μM) to prepare a series of solutions. Samples were excited with a picosecond pulse diode laser (200 kHz) at 370 nm. The emission was collected at 620 nm using an Edinburgh Instruments OD470 single-photon counting spectrometer and a high-speed red detector. A long pass filter (570 nm) was added to avoid scattering. Inner filter effect was not corrected since Aβ does not absorb at 370 nm, and it is known that correction is not necessary for moderate levels of scattering.^21^ The lifetimes and preexponential factors for the different samples were determined using global fitting of all the decay curves.

### Real-time aggregation assays using lifetime

Aβ aliquots were taken at different incubation times and mixed with [Ru(bpy)_2_(dpqp)]^2+^ in aqueous solution to a final concentration of 50 μM and 3 μM, respectively. Time-resolved decays were collected for 1200 s for all samples to make the total photoluminescence intensity consistent between samples. The lifetime was fitted with a bi-exponential decay where *τ*_1_ was fixed to be the lifetime of free complexes. In addition, 20 μM ThT was added to each sample after lifetime measurements, allowing the detection of Aβ fibrils.

### Computational simulations

The initial structure of [Ru(bpy)_2_(dpqp)]^2+^ was modeled using the [Ru(bpy)_2_(dppz)]^2+^ - DNA co-crystal structure (PDB ID: 4E1U).^22^ The structure was optimized at the B3LYP^23^/LANLTZ (+)^24^ level of theory utilizing the Hay–Wadt effective core potential^25^ for Ru^2+^ using the Gaussian 09 program^26^. The rest of the atoms (C, N, H) were treated with the 6–31 g(d) basis set. Additionally, dispersion effects were included utilizing the Grimme's function with the Becke-Johnson damping effect (GD3BJ).^27^ The initial [Ru(bpy)_2_(dpqp)]^2+^ - Aβ fibril complex was built through molecular-docking procedures on the two-fold Aβ_1–40_ fibril structure provided by Robert Tycko^28^ using the Autodock Vina 1.5.6 software^29^ (Fig. S1 and S2†). In the docking process, the Ru^2+^ was replaced by Fe^2+^ since the parameters for Ru were not available in the program. This approximation was not expected to affect the binding poses, given the [Ru(bpy)_2_(dpqp)]^2+^ complex interacts with the Aβ fibril only through its dpqp and bpy aromatic ligands.

The energetically most stable poses of the [Ru(bpy)_2_(dpqp)]^2+^- Aβ complex provided by the docking procedure were equilibrated through 200 ns all-atom MD simulations using the AMBER 03 force field as implemented in the GROMACS program^30,31^ in explicit aqueous solution. This complex was placed in cubic boxes (80 × 80 × 80 Å) filled with TIP3P^32^ water molecules. Sodium and chloride ions were added to simulate a physiological concentration of 0.154 mM. A matrix of distance restraints (semi-rigid model) with a high energetic penalty (≥1000 kJ mol^−1^) was added to the [Ru(bpy)_2_(dpqp)]^2+^ complex in order to accurately maintain the geometry as well as the coordination to the metal center. Parameters for ruthenium were obtained from a previous experimental work done with a similar kind of ligand environment.^11^ The root mean square deviation (RMSD) of the trajectories for sites 1 and 2 are shown in Fig. S3.† Analysis of the trajectories and simulated structures were performed with the inbuilt tools of the GROMACS program. The binding free energies between the Aβ_40_ fibril and [Ru(bpy)_2_(dpqp)]^2+^ complexes were calculated using a thermodynamic cycle that defines the bound and unbound states applying the lambda (*λ*) particle approach.^33,34^ The dependence of these structures on force field parameters and water models is further assessed by performing MD simulations with AMBER 99-ILDN force field^35^ and TIP4P-EW water model^36^. The structures provided by 50 ns production phase of these simulations were subsequently used to compute binding energies using the molecular mechanics/Poisson–Boltzmann surface area (MM/PBSA) method.^37^

## Results and discussion

**Fig. 1:**
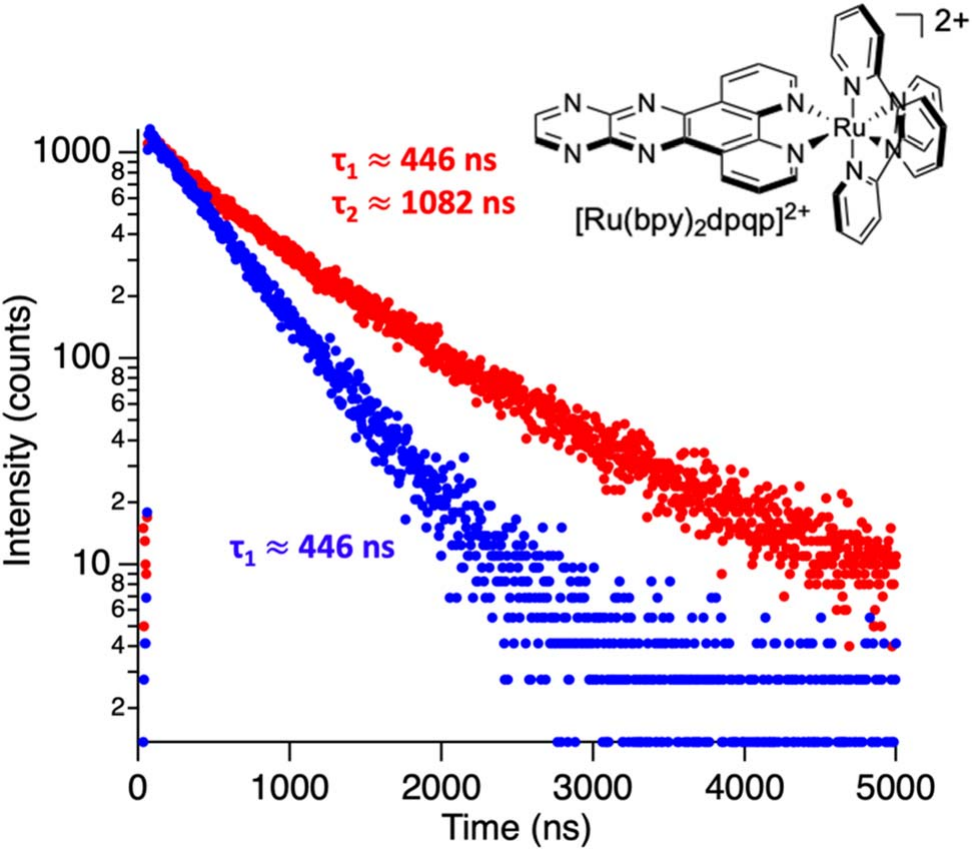
Photoluminescence decay of [Ru(bpy)_2_(dpqp)]^2+^ in aqueous solution (blue) and in an aqueous suspension of Aβ fibrils (red).

Photoluminescence lifetime is an intrinsic property of an emissive probe, which represents the average time a molecule spends in the excited state before emitting a photon. The photoluminescence decay can be expressed as: 1
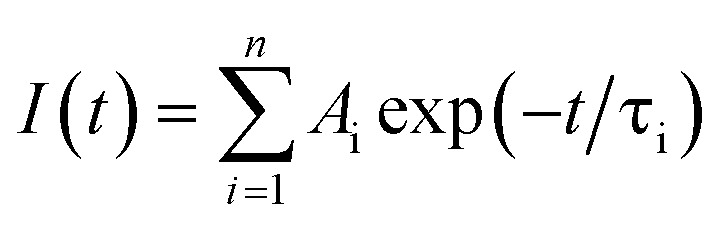
where *A*_i_ is the photoluminescence intensity at time zero (also called the preexponential factor or amplitude) and *τ*_i_ is the lifetime of the i^th^ component. The excited state decay of a photoluminescent molecule in dilute solution of a pure solvent can be typically described with a single exponential (*i* = 1 in eqn (1)). However, when more complex environments are present, then multiple exponentials are needed to describe the time-decay curves. The photoluminescence lifetime of d6 metal polypyridyl complexes is generally in the range of tens of nanoseconds to microseconds due to a mixed singlet-triplet excited state.^38–46^ This photoluminescence is sensitive to the microenvironment around the complex. For [Ru(bpy)_2_(dpqp)]^2+^ in aqueous solution, the lifetime of the free complex is *ca.* 446 ns (shown in Fig. 1 blue curve). The lifetime of [Ru(bpy)_2_(dpqp)]^2+^ does not change significantly in the presence of Aβ monomers (Fig. S4†). We found that in the presence of Aβ fibrillar aggregates, the photoluminescence decay curves show a bi-exponential decay profile with lifetimes of *ca.* 446 ns and *ca.* 1082 ns (Fig. 1, red curve). The fastest component is assigned to [Ru(bpy)_2_(dpqp)]^2+^ free in solution, and the slow component is related to [Ru(bpy)_2_(dpqp)]^2+^ bound to Aβ fibrils. This significant increase in photoluminescence lifetime is selective for Aβ fibrils and not observed for oligomers or monomers, allowing the use of [Ru(bpy)_2_(dpqp)]^2+^ as a probe for detecting Aβ fibril formation and binding sites. This is because the lifetime and preexponential factors are proportional to the emission intensity of each component and ultimately to the concentration of the free and Aβ-bound species. The total photoluminescence intensity (for a two lifetimes decay) can be determined by integrating the decay law (eqn (1)) as: 2
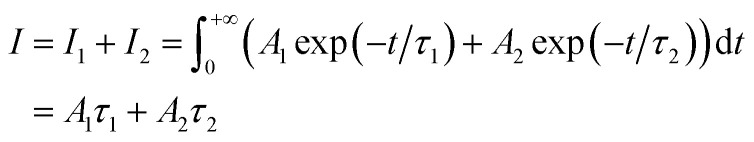
where *I*_1_ and *I*_2_ are the intensities of component 1 and 2, *A*_1_ and *A*_2_ are the amplitudes of component 1 and 2, and *τ*_1_ and *τ*_2_ are the lifetimes of component 1 and 2, respectively.

**Fig. 2:**
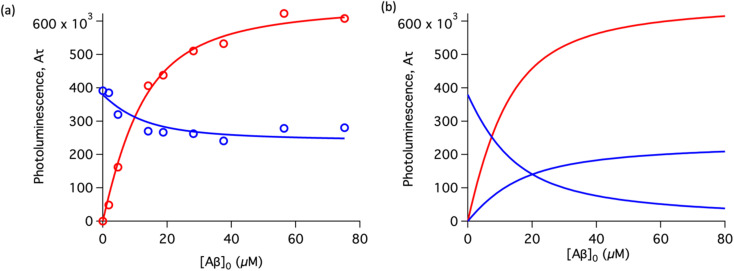
Binding equilibrium experiments. (a) Titration curves showing the different components of the decay of [Ru(bpy)_2_(dpqp)]^2+^ with different concentrations of Aβ fibrils. The red circles represent the intensity of the component with *ca.* 1082 ns lifetime. The blue circles represent the intensity of the component with *ca.* 446 ns lifetime. Red and blue solid lines represent non-linear least-square fits to eqn (11) and (12) respectively, fitted simultaneously using the same floating parameters. (b) Simulated emission of [Ru(bpy)_2_(dpqp)]^2+^ bound to site 1 (increasing red curve), site 2 (increasing blue curve), and free in solution (decreasing blue curve) using eqn (1) and the data in Table S1a.† The sum of both blue curves in (b) reproduces the blue curve in (a).

To study the interaction of [Ru(bpy)_2_(dpqp)]^2+^ and Aβ, we performed a titration experiment where the concentration of [Ru(bpy)_2_(dpqp)]^2+^ is held constant and the integrated photoluminescence is plotted as a function of the concentration of Aβ fibrils. Interestingly, the emergence of the 1082 ns component can be tracked clearly (Fig. 2a red curve) and shows a behavior similar to previous amyloid binding dyes that have been fitted to the one-site model below:3
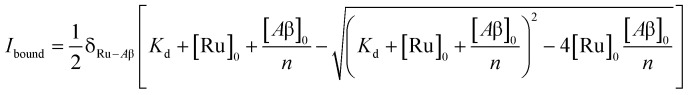
where, *δ*_Ru−Aβ_ is the proportionality constant that relates the photoluminescence signal and the concentration of [Ru(bpy)_2_(dpqp)]^2+^, *K*_d_ is the dissociation constant, [Ru]_0_ and [Aβ]_0_ are the total concentrations of [Ru(bpy)_2_(dpqp)]^2+^ and Aβ fibrils respectively, and *n* is the number of Aβ monomers that come together to form the binding site. A one-site model implies that as the 1082 ns lifetime component increases in intensity, the 446 ns component would decrease proportionally. However, when we examine the intensity of the 446 ns component, the lifetime seems to decrease initially before reaching to a steady-state and not decaying to zero. While the 1082 ns component intensity seems to be leveling off near 80 μM Aβ fibrils (shown in Fig. 2a, red curve), the 446 ns component still shows more than 60% of its original intensity (shown in Fig. 2a, blue curve). This behavior is completely unexpected and only observable due to the ability of time-resolved photoluminescence spectroscopy to provide information about the different species present. This new information led us to conclude that a different binding model is necessary to explain the data in Fig. 2a.

The behavior of the data in Fig. 2a can be explained by considering an additional binding site (site 2) that would allow the binding of [Ru(bpy)_2_(dpqp)]^2+^ to Aβ without influencing its lifetime. This is in contrast with what happens when the probe binds to site 1, which promotes an increase in lifetime to *ca.* 1082 ns. Due to the relative rigidity of the Aβ fibril's backbone (in comparison with well-folded proteins), we will assume these binding sites are independent of each other. For a two-site non-cooperative binding model, we will use the expression from Wang & Jiang,^47^ as implemented by Brautigam and co-workers:^48^4
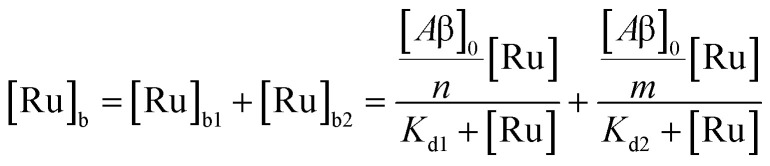
where [Ru]_b_ is the total concentration of [Ru(bpy)_2_(dpqp)]^2+^ bound to Aβ, [Ru]_b1_ and [Ru]_b2_ represent the concentration of [Ru(bpy)_2_(dpqp)]^2+^ bound to sites 1 and 2 respectively, *n* and *m* represent the number of monomers making up binding sites 1 and 2 respectively, *K*_d1_ and *K*_d2_ are the dissociation constants for sites 1 and 2 respectively, [Ru] is the concentration of free [Ru(bpy)_2_(dpqp)]^2+^, and [Aβ]_0_ is the total Aβ concentration. By substituting the mass conservation equation in (4) we get:

5 [Ru]^3^ + *a*[Ru]^2^ + *b*[Ru] + *c* = 0

where,6
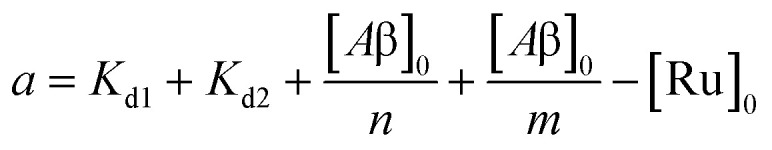
7
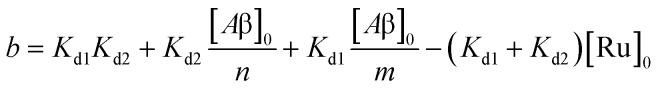

8 *c* = −*K*_d1_*K*_d2_[Ru]_0_

where [Ru]_0_ is the total concentration of [Ru(bpy)_2_(dpqp)]^2+^. This equation yields only one physically meaningful root:^47^9
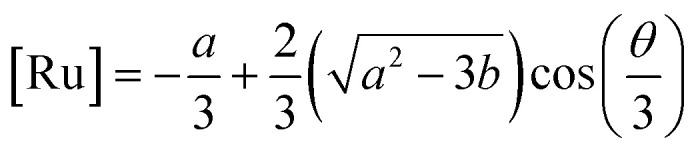
10
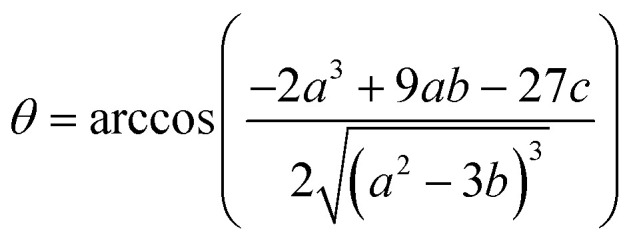
Combining (9) with mass action equations, it is possible to derive a mathematical model that fits the data in Fig. 2. The saturation curve for the 1082 ns component can be fitted to (see ESI† appendix 1 for details):11
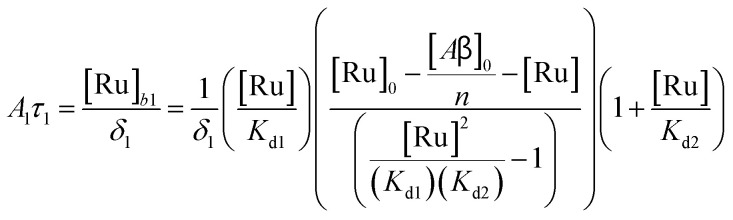
where *δ*_1_ is a proportionality constant that relates the intensity of the 1082 ns component with its concentration and [Ru] is given by (9). Similarly, the saturation curve for the 446 ns component (composed of free and bound ligand) can be fitted to:12
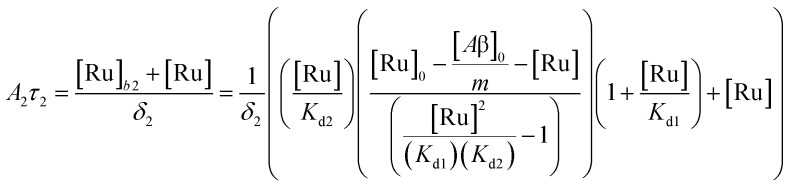
where *δ*_2_ is a proportionally constant that relates the intensity of the 446 ns component with its concentration and [Ru] is given by (9). Here, since the lifetime of [Ru(bpy)_2_(dpqp)]^2+^ does not change between free in solution *versus* bound to site 2, we make the reasonable assumption that the quantum yield of free and bound [Ru(bpy)_2_(dpqp)]^2+^ is similar and a single *δ*_2_ value is adequate to describe the trend. While (11) and (12) could be fit independently to *A*_1_*τ*_1_ and *A*_2_*τ*_2_ respectively, the real challenge is to fit the two sets of data with two equations simultaneously, while minimizing the parameters *K*_d1_, *K*_d2_, *δ*_1_ and *δ*_2_. Parameters *n* and *m* have been selected to be 5 for simplicity and implies a single ruthenium complex is bound to 5 Aβ monomers (see MD simulation below for rationale). Fig. 2a, S5a and b† shows curve fits of the data, and Table S1† displays the obtained parameters. Table 1 shows the averages and standard deviations of these three sets of experiments. Using the data in Table S1a,† we simulated [Ru(bpy)_2_(dpqp)]^2+^ binding to site 1, site 2, and free in solution at different concentrations of Aβ (Fig. 2b). The two blue curves, one increasing and one decreasing, represent the binding of [Ru(bpy)_2_(dpqp)]^2+^ to site 2 and the disappearing of free [Ru(bpy)_2_(dpqp)]^2+^ as the Aβ concentration increases, respectively. The sum of the two blue curves in Fig. 2b, yield the blue curve in Fig. 2a.

**Table 1:** Averaged values of parameters for the binding of [Ru(bpy)_2_(dpqp)]^2+^ to Aβ fibrils described by a non-cooperative two-sites model

Parameter	Values^a^
*K*_d1_	3 ± 1 μM
*K*_d2_	2.2 ± 0.5 μM
*δ*_1_	(2.6 ± 0.4) × 10^−6^ M
*δ*_2_	(10.9 ± 0.5) × 10^−6^ M

a The two data sets in Fig. 2a, S5a and b were simultaneously fit to eqn (11) and (12) (global fit) to obtain the dissociation constants and proportionality constants.

**Fig. 3:**
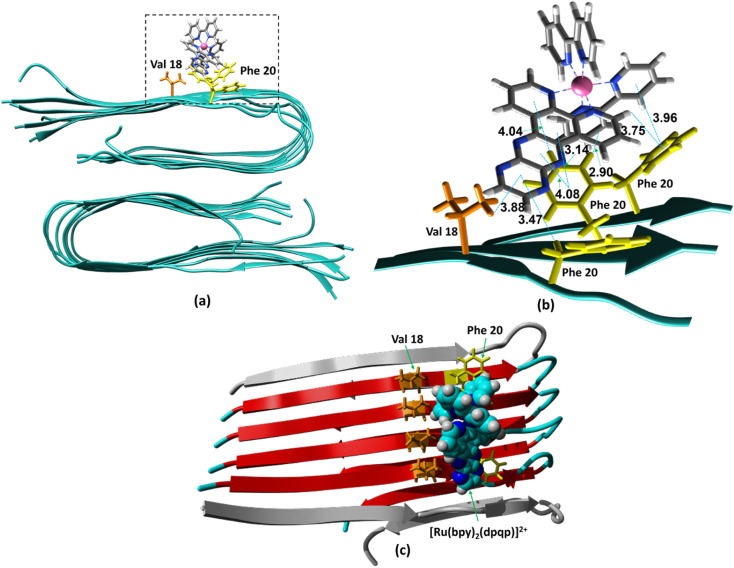
Binding of [Ru(bpy)_2_(dpqp)]^2+^ to Aβ site 1 by MD simulations. (a) The most representative structure of [Ru(bpy)_2_(dpqp)]^2+^ bound to a 2-fold Aβ_9–40_ fibril after MD simulations, and (b) zoomed view showing binding interactions. (c) The projection of the [Ru(bpy)_2_(dpqp)]^2+^ complex on the Aβ axis (the red colored peptides represent the projection).

**Fig. 4:**
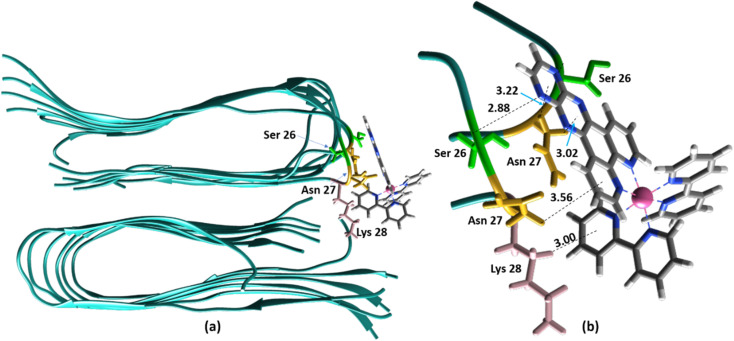
Binding of [Ru(bpy)_2_(dpqp)]^2+^ to the site 2 of Aβ by MD simulations; (a) the most representative structure of [Ru(bpy)_2_(dpqp)]^2+^ bound to a 2-fold Aβ_9–40_ fibril after MD simulations and (b) zoomed view showing binding interactions at site 2.

The ability to extract the different species contributing to an emission signal has led us to propose the existence of two different binding sites on Aβ: site 1 that affects the lifetime of [Ru(bpy)_2_(dpqp)]^2+^ and site 2, which shows a smaller dissociation constant and does not present a change in lifetime upon binding to Aβ. To investigate the nature of these binding sites, we combined molecular docking with all-atom MD simulations. We observed in the energetically stable poses (Fig. S1 and S2†) provided by the docking procedure that the metal complex was bound to the Val18-Phe20 region (Fig. 3) and on the surface near Asn27 on the Aβ fibril (Fig. 4). In the Val18-Phe20 site, the bpy and dpqp ligands of the metal complex associate with the fibrils through hydrophobic interactions. In detail, both bpy and dpqp ligands interact with the side chains of multiple Phe20 *via* CH–π and π–π interactions, and the aromatic ring of dpqp ligand interacts with the side chain of Val18 *via* CH–π interaction. This binding site largely remained unchanged throughout the 200 ns MD simulations in explicit aqueous solution (Fig. S3†). In the equilibrated structure, there was only a slight shift of the metal complex towards Phe20 residues (Fig. 3a). In particular, both CH–π and π–π interactions between the dpqp ring and Phe20 became stronger, while CH–π interaction with the side chains of Val18 got weaker (Fig. 3b). At this site, the dpqp ligand is deeply buried in the hydrophobic cleft of the fibril, whereas both bpy fragments are located on the opposite side towards the solvent (Fig. 3c). This binding site was similar to the one proposed for the binding of [Ru(bpy)_2_(dppz)]^2+^ ^11^ and [Re(CO)_3_(dppz)(Py)]^+^ ^16^ to the Aβ fibril. In this site, the [Ru(bpy)_2_(dpqp)]^2+^ complex was found to interact with at least four Aβ monomers. The same site was provided by simulations performed using the AMBER 99-ILDN/TIP4P-EW method (Fig. S6a†). For the plots in Fig. 2, we used one more Aβ monomer to establish the binding sites (*n* = *m* = 5) in order to provide a minimum separation between consecutive metal complexes for fittings to eqn (11) and (12). Given the strong change in polarity around the dpqp ligand, and knowing that the [Ru(bpy)_2_(dpqp)]^2+^ excited state is localized in the dpqp ligand, binding to this site is consistent with the change in the lifetime of [Ru(bpy)_2_(dpqp)]^2+^. It is noticeable that the dissociation constant calculated for [Ru(bpy)_2_(dpqp)]^2+^ (Table 1, *K*_d1_) is larger than that calculated for [Ru(bpy)_2_(dppz)]^2+^ ^11^ and [Re(CO)_3_(dppz)(Py)]^+^^16^. This is consistent with the larger polarity of the dpqp ligand in comparison with dppz, which leads to a weaker binding to the Val18-Phe20 hydrophobic pocket (and therefore larger dissociation constants).

Alternatively, for the other site (site 2), the backbone of Ser26 shows H bonds with the N atom on the dpqp ring. Here, the ruthenium complex is surrounded by hydrophilic amino acids such as Ser, Asn and Lys, which is likely the reason why its lifetime is barely affected. Previously, we proposed this hydrophilic binding site for [Ru(bpy)_2_(dppz)]^2+^, but it was discarded at the time because the location of amino acids 1–8 made this site less accessible to [Ru(bpy)_2_(dppz)]^2+^.^11^ It must be noted that amino acids 1–8 assume a random coil conformation and are not present in the model used.^28,49,50^ Simulations on this truncated peptide (Aβ_9–40_) using two different force fields and water models provide similar structures for this binding site (Fig. S6b†). The binding free energy (BFE) computed utilizing λ-dynamics of this site is −18.4 kcal mol^−1^, which is 11.0 kcal mol^−1^ more favorable than the one computed for site 1 (Table S2†), and can compensate for the steric constraints of amino acids 1–8. The BFE calculated through the MM/PBSA method for the AMBER 03/TIP3P and AMBER 99-ILDN/TIP4P-EW methods is also favorable by −16.7 and −11.8 kcal mol^−1^, respectively, in comparison to the site 1. This difference in BFE is caused by stronger hydrogen bonding at this site in comparison to the interactions at site 1. Interestingly, the more negative BFE value observed for site 2 agrees qualitatively with the smaller dissociation constant obtained experimentally for this site. However, quantitatively, the calculated BFE values indicate that binding to site 2 is more than twice as favorable as site 1, while the difference between the calculated *K*_d1_ and *K*_d2_ are small. The larger than expected *K*_d2_ can be explained due to the presence of amino acids 1–8 in the real peptide that is expected to partially block the access to Ser26, Asn27, and Lys28 reducing the affinity of site 2 to [Ru(bpy)_2_(dpqp)]^2+^.

**Fig. 5:**
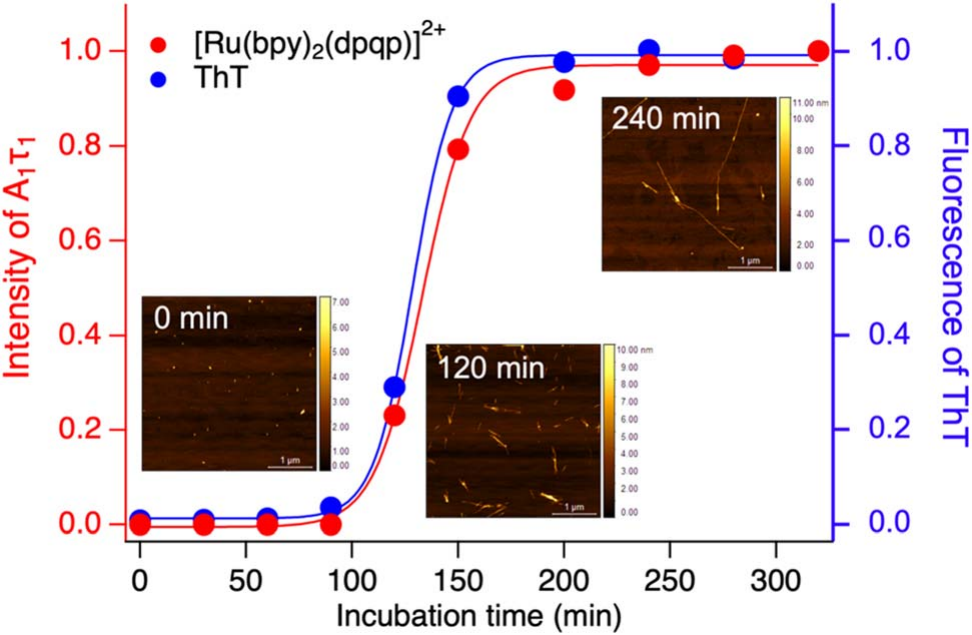
Monitoring the formation of Aβ fibrils using photoluminescence lifetime (red circle) and ThT fluorescence (blue circle). Inserted images are the AFM images of samples incubated for 0, 120 and 240 minutes.

Time-resolved photoluminescence could also be used as a tool to detect Aβ aggregation in real-time. For this, we incubated Aβ at 37 °C with orbital shaking at 600 rpm and analyzed aliquots at different time points using time-resolved spectroscopy. The photoluminescence decay curves for [Ru(bpy)_2_(dpqp)]^2+^ were obtained at different incubation times. The lifetime decay curves were fitted to eqn (1) (biexponential decays were only used when monoexponential decays did not satisfactorily fit the data), allowing for the photoluminescent intensity of [Ru(bpy)_2_(dpqp)]^2+^ bound to site 1 to be selectively determined (eqn (2)). The real-time assay is shown in Fig. 5 and displays a typical sigmoidal curve for Aβ aggregation. The intensity is low during the lag phase (0–100 minutes) where fibril growth does not take place, and AFM shows only small spherical structures, which correspond to Aβ monomers and oligomers. In this lag phase, [Ru(bpy)_2_(dpqp)]^2+^ is in the presence of soluble Aβ (which could contain monomers and oligomers),^15^ but there was no observed difference in lifetime. This is followed by a phase where fibrils are formed and elongated (propagation) which occurs from 100–320 minutes. AFM images obtained from samples at 120 minutes incubation, shortly after the lag phase, showed some short Aβ fibrils. A saturation phase is reached after 240 minutes where most Aβ has been converted to fibrils. AFM of the 240 minutes incubation sample displays abundant long Aβ fibrils. Parallel experiments done by measuring the fluorescence of ThT in the same samples show good agreement between the ThT fluorescence and the time-resolved deconvolution of the [Ru(bpy)_2_(dpqp)]^2+^. The extracted halftime of aggregation from the sigmoidal curves in Fig. 5 were 128 min and 133 min for ThT fluorescence measurement and [Ru(bpy)_2_(dpqp)]^2+^ lifetime measurement, respectively. This corroborates that both probes respond similarly to the aggregation of Aβ.

**Fig. 6:**
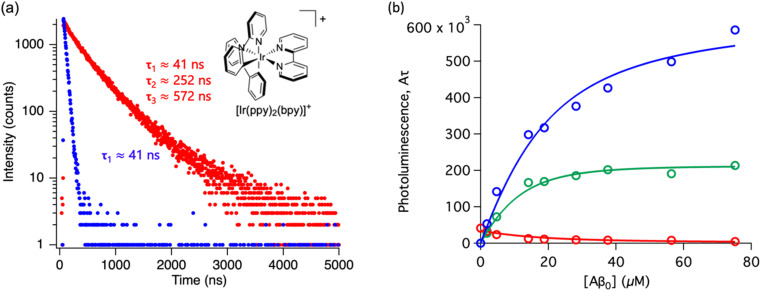
Photoluminescence lifetime of [Ir(ppy)_2_(bpy)]^+^ with Aβ fibrils. (a) Time decay curves of [Ir(ppy)_2_(bpy)]^+^ in aqueous solution (blue) and in the presence of Aβ fibrils (red). (b) Titration curve showing the different components of the decay curves of [Ir(ppy)_2_(bpy)]^+^ with different concentrations of Aβ fibrils. The red circles represent the intensity of the component with *ca.* 41 ns lifetime. The blue circles represent the intensity of the component with *ca.* 572 ns lifetime. The green circles represent the intensity of the component with *ca.* 252 ns. Curves are fits to eqn (S27)–(S29).†

Throughout this paper, we have shown that time-resolved spectroscopy can be used as a tool to extract information on molecular binding not accesible through conventional steady-state spectroscopy. To consolidate these observations, we explored two common polypyridine metal complexes without a conventional Aβ binding element, for example, dppz or dpqp. We used commercially available [Ru(bpy)_3_]^2+^ and synthesized [Ir(ppy)_2_(bpy)]^+^, and obtained the time-resolved decays as a function of the concentration of Aβ (ESI Fig. S7a† and 6a respectively). [Ru(bpy)_3_]^2+^ showed a 380 ns lifetime in air-equilibrated aqueous solution. Similar to [Ru(bpy)_2_(dpqp)]^2+^, the lifetime decay of [Ru(bpy)_3_]^2+^ became biexponential with a second component of 774 ns when Aβ nanofibrils were added (Fig. S7a†). The photoluminescence intensities of the different components (obtained from eqn (2)) as a function of Aβ concentration were plotted in Fig. S7b.† The intensities of the 380 ns component did not reach zero, similar to the short lifetime component of [Ru(bpy)_2_(dpqp)]^2+^. Therefore, we fitted the data with the two-binding site model based on eqn (11) and (12). The resulting dissociation constants for 774 ns and 380 ns were 21 μM and 4.6 μM, respectively (Table S3a†). In the case of [Ru(bpy)_2_(dpqp)]^2+^, the dissociation constant of the longer lifetime component was significantly smaller than [Ru(bpy)_3_]^2+^. This supported that the extended π system is important for the hydrophobic interaction between Val18-Phe20 of Aβ and metal complexes. For [Ir(ppy)_2_(bpy)]^+^, the decay of the complex without Aβ was monoexponential (*ca.* 41 ns), while with Aβ, the decay shows three components with lifetimes of *ca.* 41 ns, 252 ns, and 572 ns (Fig. 6a). The latter two are due to the complex bound to Aβ. The curves in Fig. 6b were fitted to a two-binding site model based on eqn S27 to S29 resulting in dissociation constants of *ca.* 9 and 1.8 μM (Table S3b†). These experiments resulted in two unprecedented observations: (1) ruthenium and iridium complexes without an amyloid binding element can bind to Aβ, and (2) they bind to two different sites on the fibrils.

## Conclusion

In summary, our studies have shown that time-resolved photoluminescence spectroscopy can be used to deconvolute binding sites of [Ru(bpy)_2_(dpqp)]^2+^ to Aβ fibrils. The dissociation constants for each binding site can be mathematically determined and reveal specific information about [Ru(bpy)_2_(dpqp)]^2+^ bound to Aβ. By considering a two sites non-cooperative binding model, we propose the existence of two different binding sites on Aβ: one that affects the lifetime of [Ru(bpy)_2_(dpqp)]^2+^ (driven by hydrophobic interactions) and one in which the lifetime is unchanged (driven by polar interactions). MD simulations lead us to propose that these two sites are the hydrophobic cleft between Phe20 and Val18 and the fibril side nearby Asn27. We also showed that we can use [Ru(bpy)_2_(dpqp)]^2+^ and time-resolved spectroscopy to monitor the aggregation of Aβ in real-time, which was confirmed by ThT assays and AFM. Finally, we used time-resolved spectroscopy to demonstrate that the common polypyridine complexes, [Ru(bpy)_3_]^2+^ and [Ir(ppy)_2_(bpy)]^+^, can bind to Aβ. This study demonstrates that time-resolved photoluminescence spectroscopy can be a powerful tool not only for excited-state dynamics but also for sensing and uncovering binding sites, which could be hidden in steady-state photoluminescence studies. The method developed in this paper can be extrapolated to study a variety of biomolecules, potentially revealing new binding sites. In addition, these principles can be used to enhance the application of FLIM to the study of amyloid aggregates. Understanding amyloid binding sites is central to the development of Aβ-binding drugs that inhibit or quench the toxicity of Aβ to neural cells.

## Data availability

We have included most of the data used for this manuscript in the paper's body or the ESI.† Specific numeric values or files are available upon request from the authors.

## Author contributions

Conceptualization, B. J., U. U., R. P., and A. A. M.; methodology, B. J., U. U., R. P., and A. A. M.; investigation, B. J., U. U., A. A., V. M. J. A., L. S., Z. M. S. H., and K. M. W.; writing – original draft, B. J., U. U., V. M. J. A., L. S., R. P., and A. A. M.; writing – review & editing, B. J., U. U., A. A., V. M. J. A., L. S., Z. M. S. H., and K. M. W., R. P., and A. A. M.; funding acquisition, R. P. and A. A. M.; resources, R. P., and A. A. M.; supervision, R. P. and A. A. M.

## Conflicts of interest

There are no conflicts to declare.

## Supplementary Material

SC-014-D2SC05418C-s001

## References

[cit1] Stelzmann R. A., Norman Schnitzlein H., Reed Murtagh F. (1995). An English Translation of Alzheimer's 1907 Paper, “Über Eine Eigenartige Erkankung Der Hirnrinde.”. Clin. Anat..

[cit2] Hardy J. A., Higgins G. A. (1992). Alzheimer's Disease: The Amyloid Cascade Hypothesis. Science.

[cit3] Karran E., Mercken M., Strooper B. D. (2011). The Amyloid Cascade Hypothesis for Alzheimer's Disease: An Appraisal for the Development of Therapeutics. Nat. Rev. Drug Discovery.

[cit4] Sevigny J., Chiao P., Bussière T., Weinreb P. H., Williams L., Maier M., Dunstan R., Salloway S., Chen T., Ling Y., O'Gorman J., Qian F., Arastu M., Li M., Chollate S., Brennan M. S., Quintero-Monzon O., Scannevin R. H., Arnold H. M., Engber T., Rhodes K., Ferrero J., Hang Y., Mikulskis A., Grimm J., Hock C., Nitsch R. M., Sandrock A. (2016). The Antibody Aducanumab Reduces Aβ Plaques in Alzheimer's Disease. Nature.

[cit5] Mullard A. (2021). More Alzheimer's Drugs Head for FDA Review: What Scientists Are Watching. Nature.

[cit6] Gabelica V., Maeda R., Fujimoto T., Yaku H., Murashima T., Sugimoto N., Miyoshi D. (2013). Multiple and Cooperative Binding of Fluorescence Light-up Probe Thioflavin T with Human Telomere DNA G-Quadruplex. Biochemistry.

[cit7] McKinley A. W., Andersson J., Lincoln P., Tuite E. M. (2012). DNA Sequence and Ancillary Ligand Modulate the Biexponential Emission Decay of Intercalated [Ru(L)_2_dppz]^2+^ Enantiomers. Chem.–Eur. J..

[cit8] Andersson J., Fornander L. H., Abrahamsson M., Tuite E., Nordell P., Lincoln P. (2013). Lifetime Heterogeneity of DNA-Bound Dppz Complexes Originates from Distinct Intercalation Geometries Determined by Complex–Complex Interactions. Inorg. Chem..

[cit9] McKinley A. W., Lincoln P., Tuite E. M. (2013). Sensitivity of [Ru(phen)_2_dppz]^2+^ Light Switch Emission to Ionic Strength, Temperature, and DNA Sequence and Conformation. Dalton Trans..

[cit10] Silva D. E. S., Cali M. P., Pazin W. M., Carlos-Lima E., Salles Trevisan M. T., Venâncio T., Arcisio-Miranda M., Ito A. S., Carlos R. M. (2016). Luminescent Ru(II) Phenanthroline Complexes as a Probe for Real-Time Imaging of Aβ Self-Aggregation and Therapeutic Applications in Alzheimer's Disease. J. Med. Chem..

[cit11] Cook N. P., Ozbil M., Katsampes C., Prabhakar R., Martí A. A. (2013). Unraveling the Photoluminescence Response of Light-Switching Ruthenium(II) Complexes Bound to Amyloid-β. J. Am. Chem. Soc..

[cit12] Cook N. P., Torres V., Jain D., Martí A. A. (2011). Sensing Amyloid-β Aggregation Using Luminescent Dipyridophenazine Ruthenium(II) Complexes. J. Am. Chem. Soc..

[cit13] Cook N. P., Kilpatrick K., Segatori L., Martí A. A. (2012). Detection of α-Synuclein Amyloidogenic Aggregates *in Vitro* and in Cells Using Light-Switching Dipyridophenazine Ruthenium(II) Complexes. J. Am. Chem. Soc..

[cit14] Cook N. P., Archer C. M., Fawver J. N., Schall H. E., Rodriguez-Rivera J., Dineley K. T., Marti A. A., Murray I. V. J. (2013). Ruthenium Red Colorimetric and Birefringent Staining of Amyloid-β Aggregates *in Vitro* and in Tg2576 Mice. ACS Chem. Neurosci..

[cit15] Jiang B., Aliyan A., Cook N. P., Augustine A., Bhak G., Maldonado R., Smith McWilliams A. D., Flores E. M., Mendez N., Shahnawaz M., Godoy F. J., Montenegro J., Moreno-Gonzalez I., Martí A. A. (2019). Monitoring the Formation of Amyloid Oligomers Using Photoluminescence Anisotropy. J. Am. Chem. Soc..

[cit16] Aliyan A., Paul T. J., Jiang B., Pennington C., Sharma G., Prabhakar R., Martí A. A. (2017). Photochemical Identification of Molecular Binding Sites on the Surface of Amyloid-β Fibrillar Aggregates. Chem.

[cit17] Aliyan A., Kirby B., Pennington C., Martí A. A. (2016). Unprecedented Dual Light-Switching Response of a Metal Dipyridophenazine Complex toward Amyloid-β Aggregation. J. Am. Chem. Soc..

[cit18] Aliyan A., Cook N. P., Martí A. A. (2019). Interrogating Amyloid Aggregates Using Fluorescent Probes. Chem. Rev..

[cit19] Jiang B., Martí A. A. (2021). Probing Amyloid Nanostructures Using Photoluminescent Metal Complexes. Eur. J. Inorg. Chem..

[cit20] Sun Y., Collins S. N., Joyce L. E., Turro C. (2010). Unusual Photophysical Properties of a Ruthenium(II) Complex Related to [Ru(bpy)_2_(dppz)]^2+^. Inorg. Chem..

[cit21] Xu J. X., Vithanage B. C. N., Athukorale S. A., Zhang D. (2018). Scattering and Absorption Differ Drastically in Their Inner Filter Effects on Fluorescence, Resonance Synchronous, and Polarized Resonance Synchronous Spectroscopic Measurements. Analyst.

[cit22] Song H., Kaiser J. T., Barton J. K. (2012). Crystal Structure of Δ-[Ru(bpy)_2_dppz]^2+^ Bound to Mismatched DNA Reveals Side-by-Side Metalloinsertion and Intercalation. Nat. Chem..

[cit23] Becke A. D. (1993). Density-functional Thermochemistry. III. The Role of Exact Exchange. J. Chem. Phys..

[cit24] Roy L. E., Hay P. J., Martin R. L. (2008). Revised Basis Sets for the LANL Effective Core Potentials. J. Chem. Theory Comput..

[cit25] Hay P. J., Wadt W. R. (1985). Ab Initio Effective Core Potentials for Molecular Calculations. Potentials for the Transition Metal Atoms Sc to Hg. J. Chem. Phys..

[cit26] Frisch M. J. E. A. , Trucks G. W., Schlegel H. B., Scuseria G. E., Robb M. A., Cheeseman J. R., Scalmani G., Barone V., Mennucci B., Petersson G. and Nakatsuji H., Gaussian 09, Revision D. 01, Gaussian Inc, Wallingford CT, 2009, P. 201

[cit27] Johnson E. R., Becke A. D. (2006). A Post-Hartree-Fock Model of Intermolecular Interactions: Inclusion of Higher-Order Corrections. J. Chem. Phys..

[cit28] Petkova A. T., Yau W.-M., Tycko R. (2006). Experimental Constraints on Quaternary Structure in Alzheimer's β-Amyloid Fibrils. Biochemistry.

[cit29] Trott O., Olson A. J. (2010). AutoDock Vina: Improving the Speed and Accuracy
of Docking with a New Scoring Function, Efficient Optimization, and Multithreading. J. Comput. Chem..

[cit30] Berendsen H. J. C., van der Spoel D., van Drunen R. (1995). GROMACS: A Message-Passing Parallel Molecular Dynamics Implementation. Comput. Phys. Commun..

[cit31] Cornell W. D., Cieplak P., Bayly C. I., Gould I. R., Merz K. M., Ferguson D. M., Spellmeyer D. C., Fox T., Caldwell J. W., Kollman P. A. (1995). A Second Generation Force Field for the Simulation of Proteins, Nucleic Acids, and Organic Molecules. J. Am. Chem. Soc..

[cit32] Price D. J., Brooks C. L. (2004). A Modified TIP3P Water Potential for Simulation with Ewald Summation. J. Chem. Phys..

[cit33] Guo Z., Brooks C. L., Kong X. (1998). Efficient and Flexible Algorithm for Free Energy Calculations Using the λ-Dynamics Approach. J. Phys. Chem. B.

[cit34] Knight J. L., Brooks III C. L. (2009). λ-Dynamics Free Energy Simulation Methods. J. Comput. Chem..

[cit35] Lindorff-Larsen K., Piana S., Palmo K., Maragakis P., Klepeis J. L., Dror R. O., Shaw D. E. (2010). Improved Side-Chain Torsion Potentials for the Amber ff99SB Protein Force Field. Proteins Struct. Funct. Bioinforma..

[cit36] Horn H. W., Swope W. C., Pitera J. W., Madura J. D., Dick T. J., Hura G. L., Head-Gordon T. (2004). Development of an Improved Four-Site Water Model for Biomolecular Simulations: TIP4P-Ew. J. Chem. Phys..

[cit37] Kumari R., Kumar R., Lynn A. (2014). *G_mmpbsa* —A GROMACS Tool for High-Throughput MM-PBSA Calculations. J. Chem. Inf. Model..

[cit38] Martí A. A., Mezei G., Maldonado L., Paralitici G., Raptis R. G., Colón J. L. (2005). Structural and Photophysical Characterisation of Fac-[Tricarbonyl(Chloro)(5,6-Epoxy-1,10-Phenanthroline)Rhenium(I)]. Eur. J. Inorg. Chem..

[cit39] Martí A. A., Colón J. L. (2010). Photophysical Characterization of the Interactions among Tris(2,2′-Bipyridyl)Ruthenium(II) Complexes Ion-Exchanged within Zirconium Phosphate. Inorg. Chem..

[cit40] Saha A., Ghosh S., Behabtu N., Pasquali M., Martí A. A. (2011). Single-Walled Carbon Nanotubes Shell Decorating Porous Silicate Materials: A General Platform for Studying the Interaction of Carbon Nanotubes with Photoactive Molecules. Chem. Sci..

[cit41] Huang K., Bulik I. W., Martí A. A. (2012). Time-Resolved Photoluminescence Spectroscopy for the Detection of Cysteine and Other Thiol Containing Amino Acids in Complex Strongly Autofluorescent Media. Chem. Commun..

[cit42] Huang K., Martí A. A. (2012). Optimizing the Sensitivity of Photoluminescent Probes Using Time-Resolved Spectroscopy: A Molecular Beacon Case Study. Anal. Chem..

[cit43] Saha A., Panos Z., Hanna T., Huang K., Hernández-Rivera M., Martí A. A. (2013). Three-Dimensional Solvent-Vapor Map Generated by Supramolecular Metal-Complex Entrapment. Angew. Chem., Int. Ed..

[cit44] Huang K., Jiang C., Martí A. A. (2014). Ascertaining Free Histidine from Mixtures with Histidine-Containing Proteins Using Time-Resolved Photoluminescence Spectroscopy. J. Phys. Chem. A.

[cit45] Martí A. A. (2015). Metal Complexes and Time-Resolved Photoluminescence Spectroscopy for Sensing Applications. J. Photochem. Photobiol. Chem..

[cit46] Ling K., Ogle M. M., Flores E., Godoy F., Martí A. A. (2022). Exploring the Photophysical Properties of UiO-67 MOF Doped with Rhenium Carbonyl Complexes. J. Photochem. Photobiol..

[cit47] Wang Z.-X., Jiang R.-F. (1996). A Novel Two-Site Binding Equation Presented in Terms of the Total Ligand Concentration. FEBS Lett..

[cit48] Tso S.-C., Chen Q., Vishnivetskiy S. A., Gurevich V. V., Iverson T. M., Brautigam C. A. (2018). Using Two-Site Binding Models to Analyze Microscale Thermophoresis Data. Anal. Biochem..

[cit49] Petkova A. T., Ishii Y., Balbach J. J., Antzutkin O. N., Leapman R. D., Delaglio F., Tycko R. (2002). A Structural Model for Alzheimer's β-Amyloid Fibrils Based on Experimental Constraints from Solid State NMR. Proc. Natl. Acad. Sci..

[cit50] Olofsson A., Sauer-Eriksson A. E., Öhman A. (2006). The Solvent Protection of Alzheimer Amyloid-β-(1–42) Fibrils as Determined by Solution NMR Spectroscopy. J. Biol. Chem..

