# Supplementary material for: Deconvoluting binding sites in amyloid nanofibrils using time-resolved spectroscopy
Source: Chem Sci. 2023 Jan 19;14(5):1072–81. doi: 10.1039/d2sc05418c (PMC9891369; doi:10.1039/d2sc05418c)
Supplement: SC-014-D2SC05418C-s001 [file SC-014-D2SC05418C-s001.pdf]

Electronic Supplementary Material (ESI) for Chemical Science.
This journal is © The Royal Society of Chemistry 2023

Supporting Information

Deconvoluting Binding Sites in Amyloid Nanofibrils using Time-Resolved Spectroscopy

Bo Jiang,[1] Utana Umezaki,[1] Andrea Augustine,[1] Vindi M. Jayasinghe-Arachchige,[2] Leonardo F. Serafim,[2] Zhi Mei Sonia He,[1] Kevin M. Wyss,[1] Rajeev Prabhakar,[2] Angel A. Martí[1,2,4]

[1] Department of Chemistry, Rice University, Houston, TX.

[2] Department of Chemistry, University of Miami, Coral Gables, FL.

[3] Department of Bioengineering, Rice University, Houston, TX.

[4] Department of Materials Science & Nanoengineering, Rice University, Houston, TX.

Figure S1: Decay curve for $[Ru(bpy)_2(dpqp)]^{2+}$ in the presence of 50 μM soluble Aβ.…Page 2

Table S1: Parameters of the binding of $[Ru(bpy)_2(dpqp)]^{2+}$ to Aβ fibrils determined by fitting the data sets with equations 11 and 12…………………Page 3

Figure S2: Titration curves of two sets of binding equilibrium experiments……………Page 4

Table S2: Calculated binding free energies by λ-particle approach………………..….Page 5

Figure S3: (a) Docking results of the $[Ru(bpy)_2(dpqp)]^{2+}$ on the surface of 2-fold Aβ fibril. (b) Binding site 1 used for MD simulation (docking pose) ………………………………….Page 6

Figure S4: Docking results of the $[Ru(bpy)_2(dpqp)]^{2+}$ on the whole 2-fold Aβ fibril. (b) Binding site 2 used for MD simulation (docking pose). Molecules bound above and below the fibril are discarded as these binding sites are infrequent in comparison with sites 1 and 2………..Page 7

Figure S5: Per-residue root mean square deviation (RMSD) for site 1 and 2 during MD simulations..……………………………………………………………………....Page 8

Figure S6: Overlay of the binding sites after MD simulations with the AMBER 03/TIP3P and AMBER 99-ILDN/TIP4P-EW methods for (a) Site1 and (b) Site2. ……………………Page 9

Figure S7: Photoluminescence lifetime of $[Ru(bpy)_3]^{2+}$ with Aβ fibrils.…………..…....Page 10

Table S3: Parameters of the binding of $[Ru(bpy)_3]^{2+}$ and $[Ir(ppy)_2(bpy)]^{+}$ to Aβ fibrils....Page 11

Appendix 1…………………………………………………………………….Page 12-16

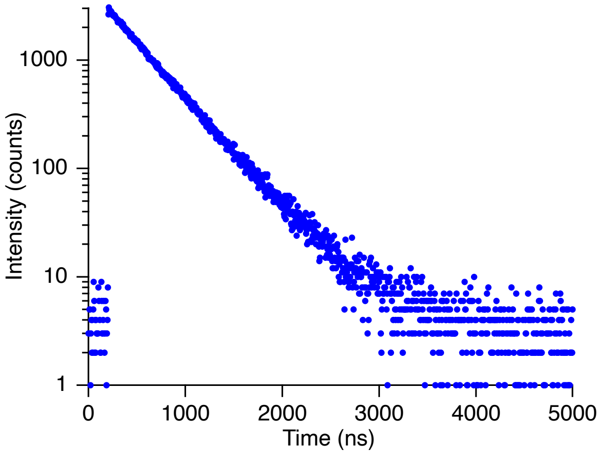

Figure S1. Decay curve for $[Ru(bpy)_2(dpqp)]^{2+}$ in the presence of 50 μM soluble Aβ.

(a)

Parameter	Values
K_{d1}	4 ± 1 μM
K_{d2}	2.5 ± 0.4 μM
δ_1	$(2.3 \pm 0.3) \times 10^{-6}$ M
δ_2	$(10.5 \pm 0.4) \times 10^{-6}$ M

(b)

Parameter	Values
K_{d1}	2.0 ± 0.4 μM
K_{d2}	1.6 ± 0.2 μM
δ_1	$(3.0 \pm 0.2) \times 10^{-6}$ M
δ_2	$(11.4 \pm 0.4) \times 10^{-6}$ M

(c)

Parameter	Values
K_{d1}	4 ± 1 μM
K_{d2}	2.4 ± 0.5 μM
δ_1	$(2.5 \pm 0.4) \times 10^{-6}$ M
δ_2	$(10.9 \pm 0.5) \times 10^{-6}$ M

Table S1. Parameters of the binding of $[Ru(bpy)_2(dpqp)]^{2+}$ to Aβ fibrils determined by fitting the data sets shown in (a) Figure 2a (b) Figure S2a (c) Figure S2b with equations 11 and 12.

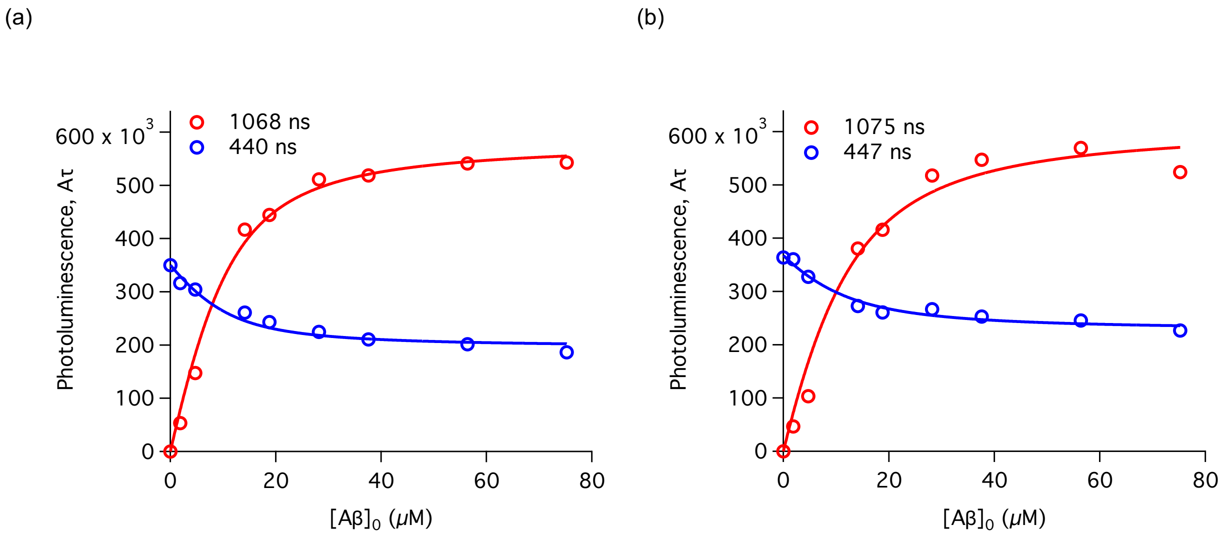

Figure S2. Titration curves of two other independent sets of binding equilibrium experiments between $[Ru(bpy)_2(dpqp)]^{2+}$ and Aβ. The red circles represent the intensity of the longer lifetime component (ca. 1082 ns). The blue circles represent the intensity of the shorter lifetime component (ca. 446 ns). Red and blue solid lines represent non-linear least-square fits to equations 11 and 12 respectively.

Binding Site	Binding Free Energy (kcal/mol)
1	-7.4
2	-18.4

Table S2. Calculated binding free energies by λ-particle approach.

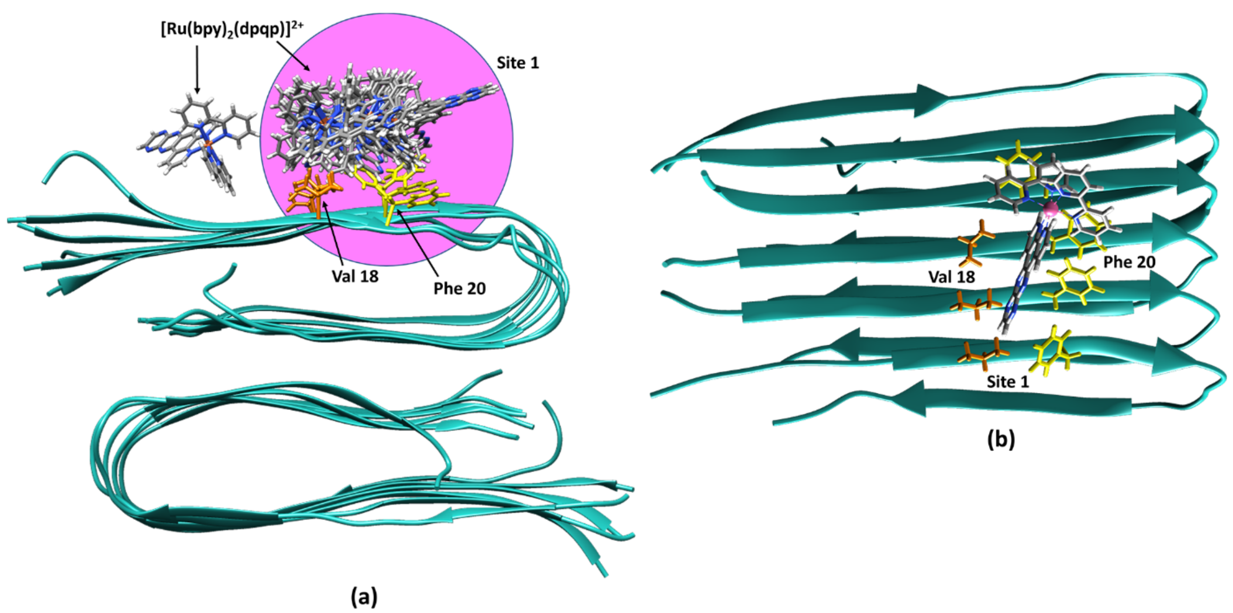

Figure S3. (a). Docking results of the $[Ru(bpy)_2(dpqp)]^{2+}$ on the surface of 2-fold Aβ fibril. (b) Binding site 1 used for MD simulation (docking pose).

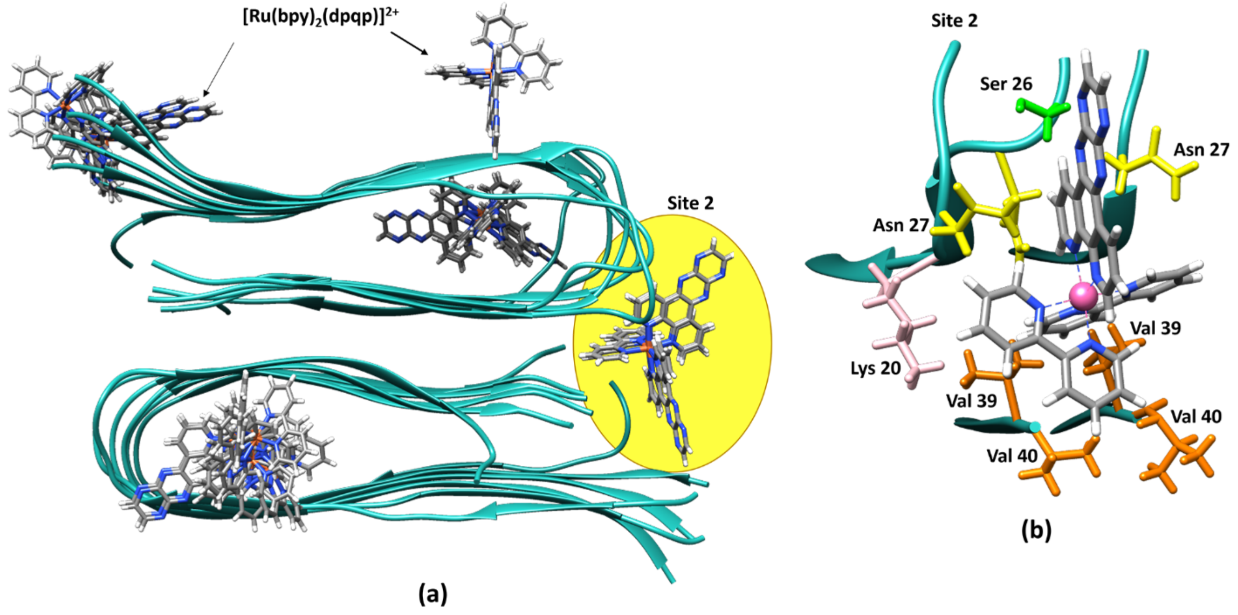

Figure S4. Docking results of the $[Ru(bpy)_2(dpqp)]^{2+}$ on the whole 2-fold Aβ fibril. (b) Binding site 2 used for MD simulation (docking pose). Molecules bound above and below the fibril are discarded as these binding sites are infrequent in comparison with sites 1 and 2.

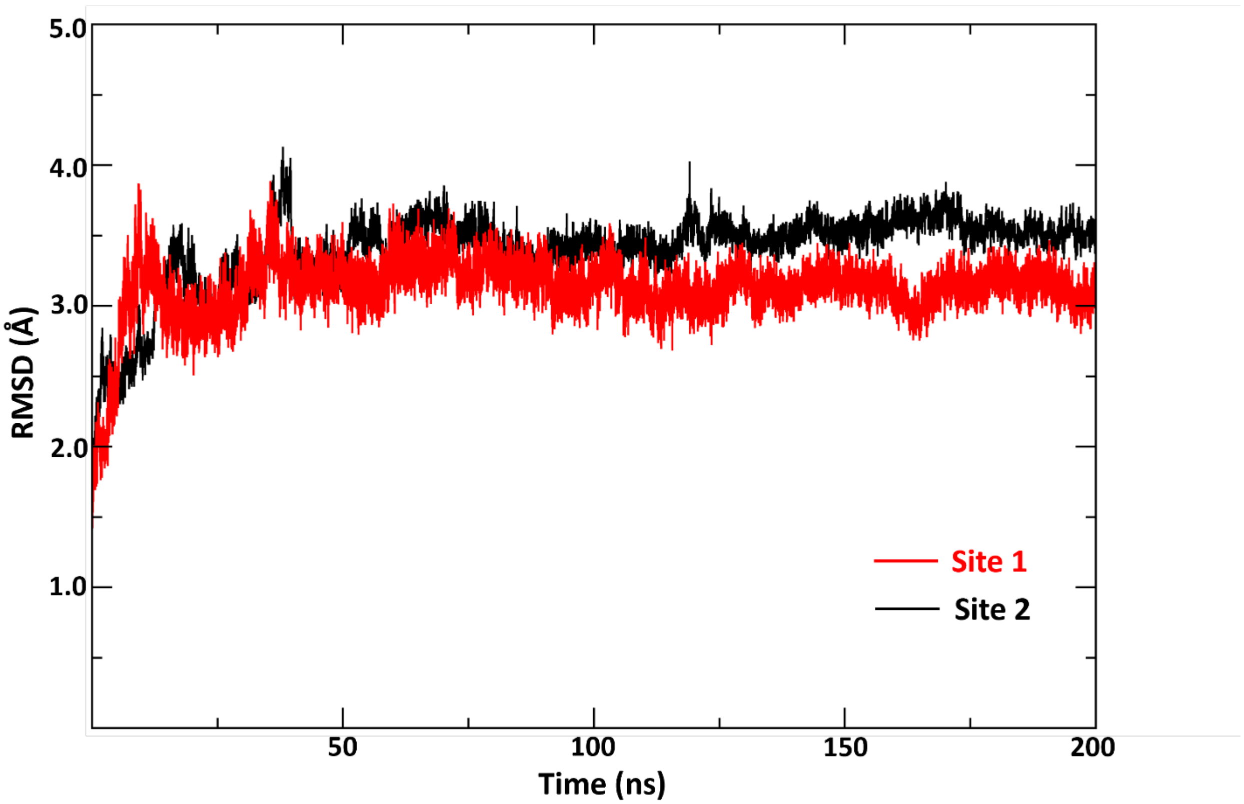

Figure S5. Per-residue root mean square deviation (RMSD) for site 1 and 2 during MD simulations.

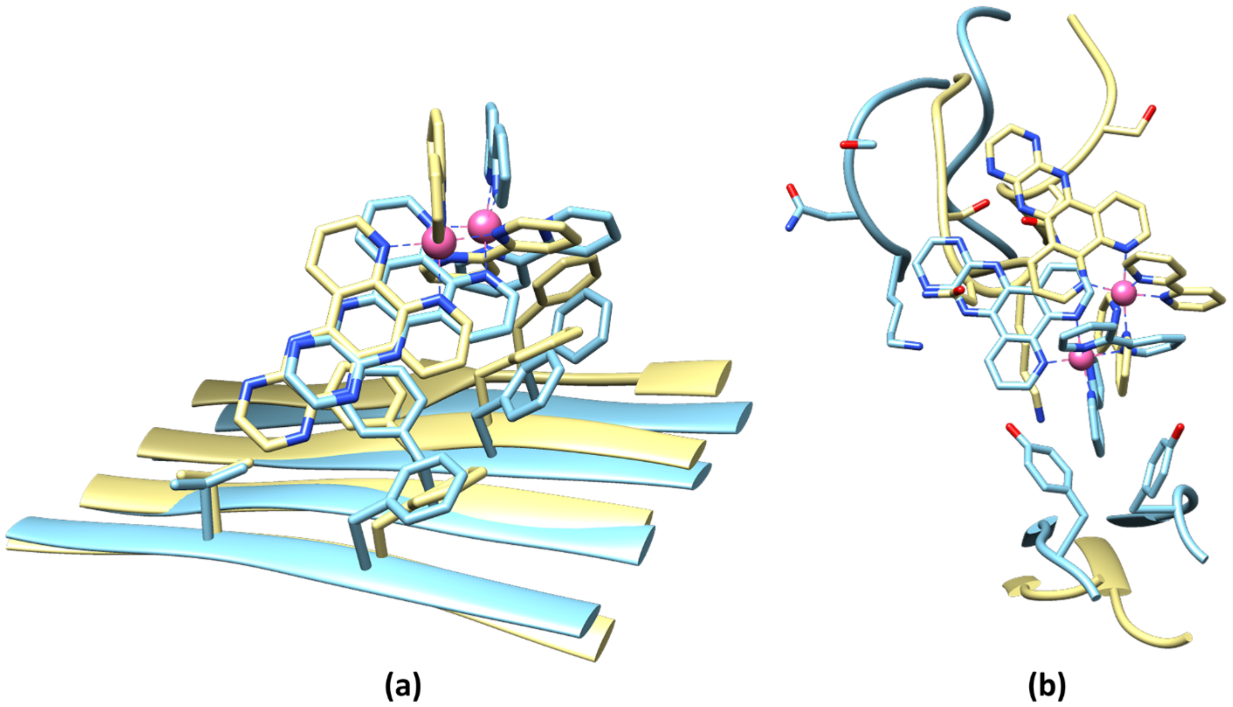

Figure S6. Overlay of the binding sites after MD simulations with AMBER 03/TIP3P (khaki color) and AMBER 99-ILDN/TIP4P-EW (sky blue color) methods for (a) Site1 and (b) Site2.

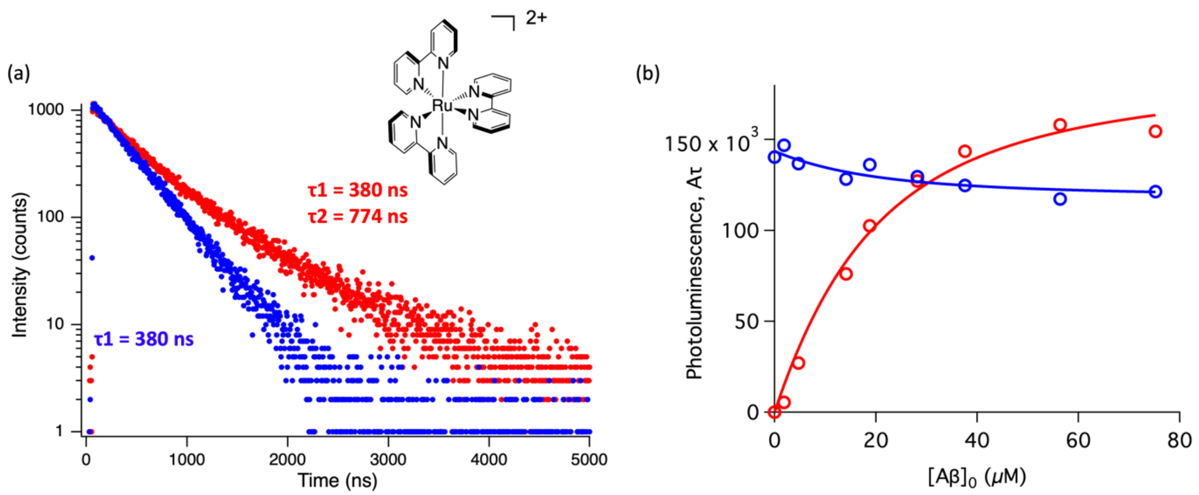

Figure S7. Photoluminescence lifetime of $[Ru(bpy)_3]^{2+}$ with Aβ fibrils. (a) Time decay curves of $[Ru(bpy)_3]^{2+}$ in aqueous solution (blue) and in the presence of Aβ fibrils (red). (b) Titration curve showing the different components of the decay curves of $[Ru(bpy)_3]^{2+}$ with different concentrations of Aβ fibrils. Curves are fits to equations 11 and 12.

(a)

Parameter	Values
K_{d1}	21 ± 6 μM
K_{d2}	4.6 ± 0.6 μM
δ_1	$(3.8 \pm 0.9) \times 10^{-6}$ M
δ_2	$(27.9 \pm 0.7) \times 10^{-6}$ M

(b)

Parameter	Values*
K_{d1}	9 ± 1 μM
K_{d2}	1.8 ± 0.3 μM
δ_1	$(157 \pm 70) \times 10^{-6}$ M
δ_2	$(3 \pm 2) \times 10^{-6}$ M
δ_3	$(7 \pm 4) \times 10^{-6}$ M

Table S3. (a) Parameters of the binding of $[Ru(bpy)_3]^{2+}$ to Aβ fibrils were determined by fitting with equations 11 and 12. (b) Parameters of the binding of $[Ir(ppy)_2(bpy)]^+$ to Aβ fibrils were determined by fitting with equations S27-S29.

* Averaged values and standard deviations of three sets of experiments.

Appendix 1

Let us consider the following equilibria:

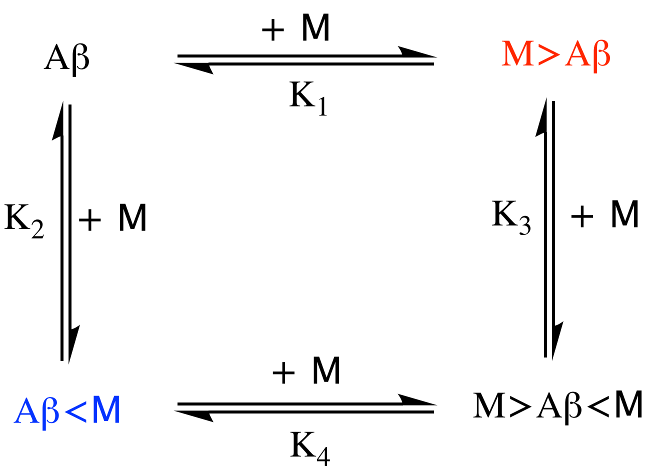

where, for two binding sites that are independent from each other:

$$K_1 = \frac{[A\beta][M]}{[M{>}A\beta]} \quad \text{(S1)}$$

$$K_2 = \frac{[A\beta][M]}{[A\beta{<}M]} \quad \text{(S2)}$$

$$K_3 = \frac{[M{>}A\beta][M]}{[M{>}A\beta{<}M]} \quad \text{(S3)}$$

$$K_4 = \frac{[A\beta{<}M][M]}{[M{>}A\beta{<}M]} \quad \text{(S4)}$$

and therefore

$$K_1 = K_4 = K_{d1} \quad \text{(S5)}$$

$$K_2 = K_3 = K_{d2} \quad \text{(S6)}$$

from the manuscript we get that the free metal complex is given by:

$$[M] = -\frac{a}{3} + \frac{2}{3}\left(\sqrt{a^2 - 3b}\right)\cos\left(\frac{\theta}{3}\right) \tag{S7}$$

$$\theta = \arccos\left(\frac{-2a^3+9ab-27c}{2\sqrt{(a^2-3b)^3}}\right) \tag{S8}$$

$$a = K_1 + K_2 + \frac{[A\beta]_0}{n} + \frac{[A\beta]_0}{m} - [M]_0 \tag{S9}$$

$$b = K_1K_2 + K_2\frac{[A\beta]_0}{n} + K_1\frac{[A\beta]_0}{m} - (K_1 + K_2)[M]_0 \tag{S10}$$

$$c = -K_1K_2[M]_0 \tag{S11}$$

From eq S1 and S5 we get:

$$K_{d1} = \frac{[A\beta][M]}{[M{>}A\beta]} \tag{S12}$$

and from eq S3 and S6 we get:

$$K_{d2} = \frac{[M{>}A\beta][M]}{[M{>}A\beta{<}M]} \tag{S13}$$

By multiplying S12 and S13 we get:

$$(K_{d1})(K_{d2}) = \frac{[A\beta][M]}{[M{>}A\beta]}\frac{[M{>}A\beta][M]}{[M{>}A\beta{<}M]} = \frac{[A\beta][M]^2}{[M{>}A\beta{<}M]} \tag{S14}$$

and solving for and solving by [M>Aβ<M] we obtain:

$$\boldsymbol{[M > A\beta < M] = \frac{[A\beta][M]^2}{(K_{d1})(K_{d2})}} \tag{S15}$$

The two mass balance equations are

$$[A\beta]_0 = [A\beta] + [M > A\beta] + [A\beta < M] + [M > A\beta < M] \quad \text{(S16)}$$

$$[M]_0 = [M] + [M > A\beta] + [A\beta < M] + 2[M > A\beta < M] \quad \text{(S17)}$$

Where [Aβ] and [M] are the concentration of free Aβ and free M.

Subtracting S17 – S16 we get:

$$[M]_0 - [A\beta]_0 = [M] + [M > A\beta < M] - [A\beta] \quad \text{(S18)}$$

Rearranging:

$$[M]_0 - [A\beta]_0 - [M] = [M > A\beta < M] - [A\beta] \quad \text{(S19)}$$

And substituting S15:

$$[M]_0 - [A\beta]_0 - [M] = \frac{[A\beta][M]^2}{(K_{d1})(K_{d2})} - [A\beta] \quad \text{(S20)}$$

Solving for [Aβ]:

$$\frac{[A\beta][M]^2}{(K_{d1})(K_{d2})} - [A\beta] = [M]_0 - [A\beta]_0 - [M]$$

$$[A\beta]\left(\frac{[M]^2}{(K_{d1})(K_{d2})} - 1\right) = [M]_0 - [A\beta]_0 - [M]$$

$$[A\beta] = \frac{[M]_0 - [A\beta]_0 - [M]}{\left(\frac{[M]^2}{(K_{d1})(K_{d2})} - 1\right)} \tag{S21}$$

Substituting S21 in S15 we obtain:

$$[M > A\beta < M] = \frac{([M]_0 - [A\beta]_0 - [M])([M]^2)}{\left(\frac{[M]^2}{(K_{d1})(K_{d2})} - 1\right)(K_{d1})(K_{d2})} \tag{S22}$$

Also, substituting S21 in S12 we obtain:

$$[M > A\beta] = \frac{[A\beta][M]}{K_{d1}} = \frac{\left(\frac{[M]_0 - [A\beta]_0 - [M]}{\left(\frac{[M]^2}{(K_{d1})(K_{d2})} - 1\right)}\right)[M]}{K_{d1}} \tag{S23}$$

substituting S21 in S2 we obtain:

$$[A\beta < M] = \frac{[A\beta][M]}{K_{d2}} = \frac{\left(\frac{[M]_0 - [A\beta]_0 - [M]}{\left(\frac{[M]^2}{(K_{d1})(K_{d2})} - 1\right)}\right)[M]}{K_{d2}} \tag{S24}$$

Therefore, for $[Ru(bpy)_2(dpqp)]^{2+}$ the saturation curve for the ca. 1082 ns component can be fitted to the sum of eq S15 and S12 where $[M]_{b1}$ is the concentration of $[Ru(bpy)_2(dpqp)]^{2+}$ bound to site 1:

$$A_1\tau_1 = \frac{[M]_{b1}}{\delta_1} = \frac{[M > A\beta] + [M > A\beta < M]}{\delta_1} = \frac{1}{\delta_1}\left(\frac{[A\beta][M]}{K_{d1}} + \frac{[A\beta][M]^2}{(K_{d1})(K_{d2})}\right) = \frac{1}{\delta_1}\left(\frac{[M]}{K_{d1}}\right)[A\beta]\left(1 + \frac{[M]}{K_{d2}}\right)$$

Now substituting [Aβ] for equation S21 we get:

$$A_1\tau_1 = \frac{1}{\delta_1}\left(\frac{[M]}{K_{d1}}\right)\left(\frac{[M]_0-\frac{[A\beta]_0}{n}-[M]}{\left(\frac{[M]^2}{(K_{d1})(K_{d2})}-1\right)}\right)\left(1+\frac{[M]}{K_{d2}}\right) \quad \text{(S25)}$$

where δ_1 is a proportionally constant that relates the intensity of the ca. 1082 ns component with its concentration and [M] is given by S7. Similarly, the saturation curve for the ca. 446 ns component (composed of free and bound ligand) can be fitted to:

$$A_2\tau_2 = \frac{[M]_{b2}+[M]}{\delta_2} = \frac{[A\beta < M] + [M > A\beta < M] + [M]}{\delta_2} = \frac{1}{\delta_2}\left(\left(\frac{[M]}{K_{d2}}\right)\left(\frac{[M]_0-\frac{[A\beta]_0}{m}-[M]}{\left(\frac{[M]^2}{(K_{d1})(K_{d2})}-1\right)}\right)\left(1+\frac{[M]}{K_{d1}}\right)+[M]\right) \quad \text{(S26)}$$

Here $[M]_0$ and $[A\beta]_0$ are the total concentration of the metal complex and Aβ respectively and [M] is given by equation S7. Including the binding stoichiometry requires that all values of $[A\beta]_0$ in equations S22, S23 and equations S22, S24 to be substituted for $[A\beta]_0/n$ and $[A\beta]_0/m$ where n and m are the number of monomers that form binding site 1 and 2 respectively.

For the binding or $[Ir(ppy)_2(bpy)]^+$, the following equations were used:

$$A_1\tau_1 = \frac{1}{\delta_1}[M] \quad \text{(S27)}$$

$$A_2\tau_2 = \frac{1}{\delta_2}\left(\frac{[M]}{K_{d1}}\right)\left(\frac{[M]_0-\frac{[A\beta]_0}{n}-[M]}{\left(\frac{[M]^2}{(K_{d1})(K_{d2})}-1\right)}\right)\left(1+\frac{[M]}{K_{d2}}\right) \quad \text{(S28)}$$

$$A_3\tau_3 = \frac{1}{\delta_3}\left(\frac{[M]}{K_{d2}}\right)\left(\frac{[M]_0-\frac{[A\beta]_0}{m}-[M]}{\left(\frac{[M]^2}{(K_{d1})(K_{d2})}-1\right)}\right)\left(1+\frac{[M]}{K_{d1}}\right) \quad \text{(S29)}$$
